# *In Vivo* Evaluation of Oxidative Stress Induced by Intraperitoneal Administration of Mannosylerythritol Lipid Biosurfactant in Swiss Mice

**DOI:** 10.3390/biom16020310

**Published:** 2026-02-16

**Authors:** Paulo Emilio Feuser, Ana Letícia Silva Coelho, Mariana de Melo Cardoso, Rahisa Scussel, Mírian Ívens Fagundes, Lariani Tamires Witt Titbohl, Isabela Karina Della-Flora, Ricardo Andrez Machado-de-Ávila, Paulo Cesar Lock Silveira, Debora de Oliveira, Cristiano José de Andrade

**Affiliations:** 1 Graduate Program in Chemical Engineering, Department of Chemical Engineering and Food Engineering, Federal University of Santa Catarina, Florianópolis 88040-900, SC, Brazil; paulofeuser@hotmail.com (P.E.F.); alscoelho@mail.uft.edu.br (A.L.S.C.); isa_dellaflora@hotmail.com (I.K.D.-F.); debora.oliveira@ufsc.br (D.d.O.); 2 Graduate Program in Health Science, Universidade do Extremo Sul Catarinense, Criciúma 88806-000, SC, Brazil; marianademeloc@gmail.com (M.d.M.C.); scussel.rahisa@gmail.com (R.S.); mifagundes@unesc.net (M.Í.F.); lariani.tietbohl@gmail.com (L.T.W.T.); r_andrez@yahoo.com.br (R.A.M.-d.-Á.); psilveira@unesc.br (P.C.L.S.)

**Keywords:** biosurfactant, glycolipid, mannosylerythritol lipids, acute toxicity, oxidative stress

## Abstract

Mannosylerythritol lipid-B (MEL-B) is a glycolipid whose biological properties have been widely investigated, especially in the skincare, food, and therapeutic fields. Despite this, few studies have addressed the toxicity of this glycolipid in vivo. Therefore, this work aimed to evaluate the in vivo oxidative stress induced by MEL-B in Swiss mice. MEL-B (50 and 150 mg/kg) was administered intraperitoneally at two exposure times, 24 and 72 h. Biochemical damage was quantified in the gastrocnemius, lungs, kidneys, heart, liver, and spleen. This study assessed the levels of reactive oxygen species, oxidative damage markers, antioxidant defenses, protein concentration, triglycerides, creatine kinase (CK-MB), and lactate dehydrogenase (LDH). DCF (2′,7′-dichlorofluorescein), sulfhydryl, and SOD (superoxide dismutase) levels were used to assess oxidative damage and antioxidant defenses in cells. The results indicate that MEL-B did not trigger acute toxicity in the tested animals in a systemic context. Oxidative stress was observed in the liver samples, likely due to the metabolization of MEL-B. The levels of triglycerides and of CK-MB and LDH enzymes did not present any significant alteration (*p* < 0.05), indicating that glycolipids do not trigger tissue damage. These findings open new perspectives for the safe use of MEL-B in cosmetic and medicinal products.

## 1. Introduction

Mannosylerythritol lipids (MELs) are natural glycolipids produced under submerged fermentation by *Pseudozyma* spp. and *Ustilago* spp. [1,2,3,4]. From the 2000s onwards, published studies went beyond the topic of MEL production and began to explore their biological properties. In the pharmaceutical, medical, and skincare fields, MELs have been shown to have antitumor, antimicrobial, moisturizing, and antimelanogenic properties [4,5,6,7]. Recent studies highlight the potential of MEL-A and MEL-B in the food industry, with great emphasis on their antimicrobial, preservative, and emulsifying properties [8,9,10,11].

Several in vitro studies have suggested the potential of MELs for biomedical applications [12,13,14]. Takahashi et al. [15] demonstrated the anti-inflammatory and antioxidant activity of MEL-C (Mannosylerythritol Lipid-C) over human fibroblasts after exposure of cells to oxidative stress conditions. Previous studies [5,6,12,14] showed that MEL-A (Mannosylerythritol Lipid-A) and MEL-B inhibited the exocytotic release of serotonin and histamine from RBL-2H3 cells (rat basophilic leukemia cell line) after eight hours of exposure. A similar result was observed for TNF-α (tumor necrosis factor) and leukotriene mediators after treatment of RBL-2H3 cells with MELs for four hours and for twenty minutes, respectively. Additionally, Ca^2+^, SNARE (soluble N-ethylmaleimide-sensitive factor attachment protein receptors), and MAP (mitogen-activated protein kinase) signaling pathways were modified by the action of glycolipids, evidencing their anti-inflammatory aspects [5,6,12,14]. In the skincare field, Bae et al. [16] suggest that MEL-B acts by inhibiting, at the mRNA level, the expression of tyrosinases, a melanogenic enzyme. Bae et al. [17] showed that MELs can restore the signaling pathway involved in aquaporin-3 overexpression after exposure of keratinocytes to UVA radiation.

However, an in vivo toxicity assessment of any novel potential drug is essential before further development. Cheng et al. [18] developed a berberine-loaded MEL-B nanomicelle delivery system to combat biofilm-induced *Helicobacter pylori* resistance. Their in vivo test showed that the system significantly reduced *H. pylori* levels, repaired the gastric mucosal barrier, and decreased inflammation, offering a safe nanodrug strategy for eradicating *H. pylori* biofilms. The study by Liu et al. [19] demonstrated that combining high hydrostatic pressure with the biosurfactant MEL-A has a synergistic bactericidal effect against Listeria monocytogenes. Additionally, the in vivo safety of MEL-A was confirmed in mouse models, suggesting its potential for use in the food industry. Another study combines MELs with nanocarriers and targeting ligands for controlled and targeted delivery [20]. The toxicological profiling of biosurfactant-encapsulated polymeric nanoparticles of polylactic acid–polyethylene glycol in mice found no significant differences in hematological, biochemical, or histopathological parameters between the control and treated groups, suggesting that nanoparticles are non-toxic and could provide a safe platform for future biomedical applications.

Recently, Feuser and collaborators [6] demonstrated that direct contact of MEL-B with erythrocytes promotes the lysis of these cells at a concentration of 80 µg/mL and is effective in inducing apoptosis in B16F10 cells (murine melanoma) in a dose-dependent manner. Furthermore, in vitro cytotoxicity analysis showed that MEL-B did not induce inflammatory or vasodilatation responses in healthy cells [6,21]. However, beyond these data, there is limited knowledge regarding the effect of MELs on healthy cells and tissues. In this regard, biochemical and toxicological studies are needed to evaluate possible allergic effects and acute or chronic health complications occasioned by MEL-B.

Thus, this study involves an initial toxicology screening to identify systemic and tissue-level perturbations following MEL-B exposure, to provide a robust basis for detecting early signs of toxicity, and MEL-B’s safety for therapeutic, pharmacological, and medical applications. The animals were euthanized at 24 h and 72 h after administration, and reactive oxygen species, oxidative damage markers, antioxidant defenses, protein content, triglycerides, CK-MB, and LDH levels were evaluated in gastrocnemius, lung, kidney, cardiac, liver, and spleen tissues.

## 2. Materials and Methods

### 2.1. Materials and Reagents

MEL-B (≥95% purity) was produced by submerged fermentation using yeast-like *Pseudozyma* spp., according to the method previously described in [14]. The glycolipid was recovered from fermentation broth using liquid–liquid extraction using a Silica open column.

2′,7′-dichlorodihydrofluorescein diacetate (DCFH-DA), sodium phosphate buffer (PBS), 5,5′-dithiobis (2-nitrobenzoic acid), SOD, bovine serum albumin, and phosphomolybdic reagent (Folin phenol) were purchased from Sigma Aldrich, São Paulo, Brazil. CK-MB, LDH, and triglycerides were obtained from Liquiform and Labtest Diagnostic (Labtest^®^, São Paulo, Brazil).

### 2.2. Animals

The experiments were performed using 36 adult Swiss mice (25–30 g), 18 male and 18 female. The animals were divided into three groups: (i) a control group without MEL-B, where PBS was used instead; (ii) mice administered MEL-B at a concentration of 50 mg/kg; and (iii) mice administered MEL-B at a concentration of 150 mg/kg. MEL-B was administered intraperitoneally (IP) to the animals. Intraperitoneal administration usually leads to faster absorption when compared to other administration routes and can be used to administer large volumes of fluid safely; thus, it is a proper route of administration [22]. The doses and treatment time used in this study were selected based on our in vitro experiments in previous studies [6].

Five animals were housed per cage under controlled conditions: 22 ± 1 °C, humidity level of 55–65%, and free access to food and water. After 24 h of administration, 50% of the animals (n = 18, 9 male and 9 female) were euthanized. A similar procedure was performed after 72 h of administration (n = 18, 9 male and 9 female). The mice were euthanized by cervical dislocation, and the tissue was surgically removed, processed, and stored in a freezer at −80 °C until further analysis. All experimental protocols were approved by the research ethics committee of UNESC (Approval number 13/2020; Approval date: 7 April 2020). The doses used in this study were selected based on our pilot experiments and previous studies.

### 2.3. Analysis of Biochemical Parameters

#### 2.3.1. Preparation of Samples for Biochemical Analyses

The tissues/organs used for the biochemical study were surgically removed and included the region of the gastrocnemius, liver, lungs, kidneys, heart, and spleen. The material obtained was homogenized in PBS, centrifuged (1000 rpm, 4 °C), and frozen at −80 °C. The liquid obtained from homogenization was also stored (−80 °C) for analysis. The maximum storage period was five days.

#### 2.3.2. Intracellular Determination of Reactive Oxygen Species (ROS)

Oxidized intracellular DCF levels were monitored in samples after incubation with DCFH-DA. The formation of the oxidized fluorescent derivative was monitored at excitation and emission wavelengths of 488 and 525 nm, respectively, using a SpectraMax^®^ (Molecular Devices, San Jose, CA, USA) fluorescence spectrophotometer [23].

#### 2.3.3. Determination of Oxidative Damage Marker Levels

The total thiol content was used to determine levels of oxidative damage markers using a 5,5′-dithiobis (2-nitrobenzoic acid) reagent. The incorporation of the compound was measured at 412 nm using a spectrophotometer and expressed in sulfhydryl content levels [24].

#### 2.3.4. Determination of Antioxidant Defenses

Antioxidant defenses were estimated based on SOD activity and quantified based on the inhibition of adrenaline autoxidation at 480 nm [25]. The results were expressed in U SOD/mg protein.

#### 2.3.5. Protein Content

The protein content of all tissues was measured according to the protein assay protocol of Lowry [26].

#### 2.3.6. Creatine Kinase (CK-MB) and Lactate Dehydrogenase (LDH) Levels

Biochemical analyses were performed with animal blood serum. The blood was collected retro-orbitally in Eppendorf tubes. Then, the samples were centrifuged at 10,000 rpm for five minutes, and the serum obtained was stored in microcentrifuge tubes at −80 °C until analysis. The following variables were measured: triglycerides, creatine kinase activity, and lactate dehydrogenase activity. All readings and measurements were performed using a spectrophotometer (SpectraMax^®^ M3) [23,24,25].

### 2.4. Statistical Analysis

The biochemical data were statistically analyzed to assess differences between treatments. A two-way ANOVA was used for all assays (*p* < 0.05) followed by Turkey’s test as a post hoc comparison. GraphPad Prism version 8.0 (San Diego, CA, USA) was used for the analyses.

## 3. Results

**Figure 1:**
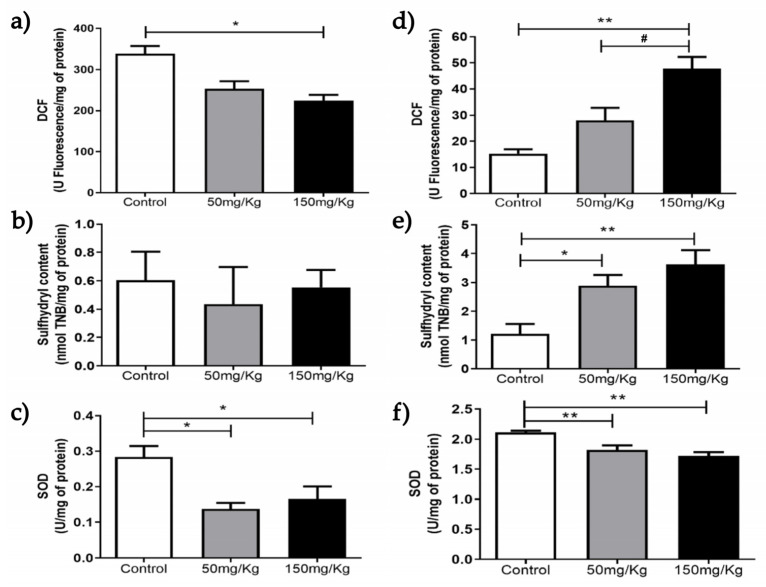
Analysis of DCF, sulfhydryl content, and SOD levels 24 h (**a**–**c**) and 72 h (**d**–**f**) after IP administration of MEL-B at concentrations of 50 mg/kg and 150 mg/kg in mouse spleens. * *p* < 0.05 and ** *p* < 0.005 when compared to the control group; ^#^ *p* < 0.05 compared to the 150 mg/kg group, according to One-way ANOVA followed by Tukey’s Multiple Comparison Test. Each group at different concentrations and time points was analyzed from n = 6 samples.

**Figure 2:**
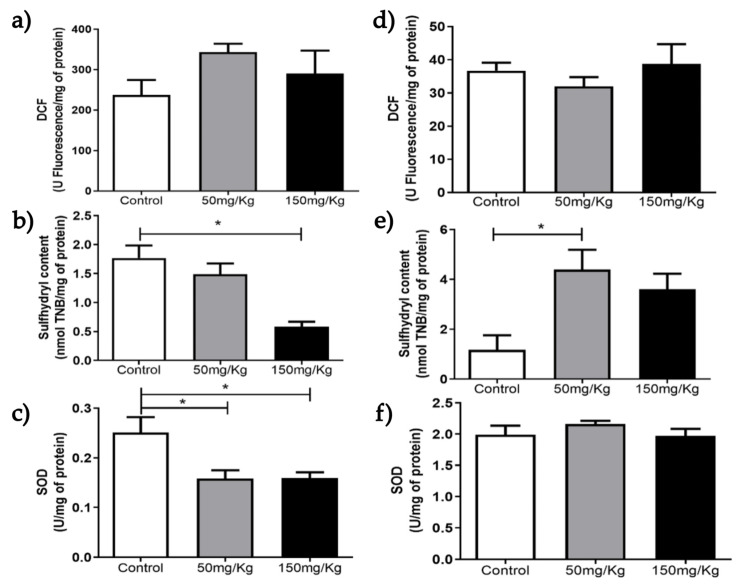
Analysis of DCF, sulfhydryl content, and SOD levels 24 h (**a**–**c**) and 72 h (**d**–**f**) after IP administration of MEL-B at concentrations of 50 mg/kg and 150 mg/kg in mouse lungs. * *p* < 0.05 when compared to the control group, according to One-way ANOVA followed by Tukey’s Multiple Comparison Test. Each group at different concentrations and time points was analyzed from n = 6 samples.

**Figure 3:**
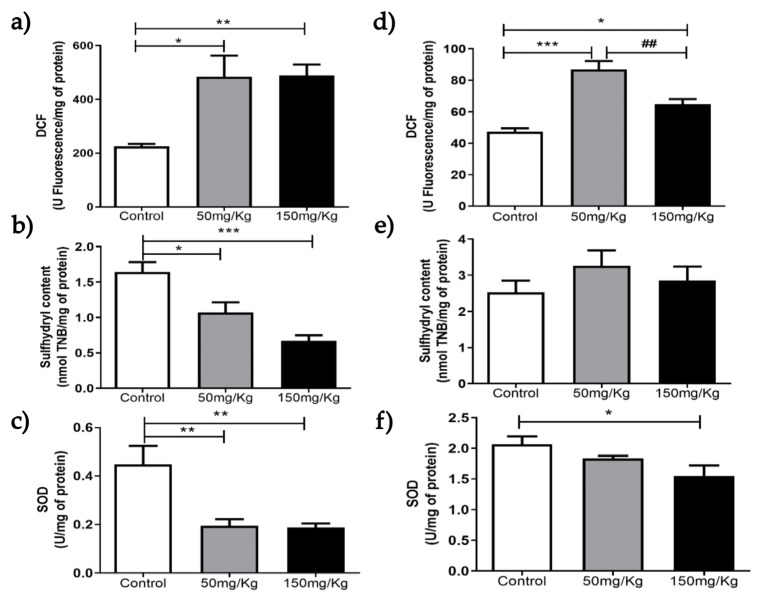
Analysis of DCF, sulfhydryl content, and SOD levels 24 h (**a**–**c**) and 72 h (**d**–**f**) after IP administration of MEL-B at concentrations of 50 mg/kg and 150 mg/kg in mouse livers. * *p* < 0.05, ** *p* < 0.005, and *** *p* < 0.0005 when compared to the control group; and ^##^ *p* < 0.005 compared to the 150 mg/kg group, according to One-way ANOVA followed by Tukey’s Multiple Comparison Test. Each group at different concentrations and time points was analyzed from n = 6 samples.

**Figure 4:**
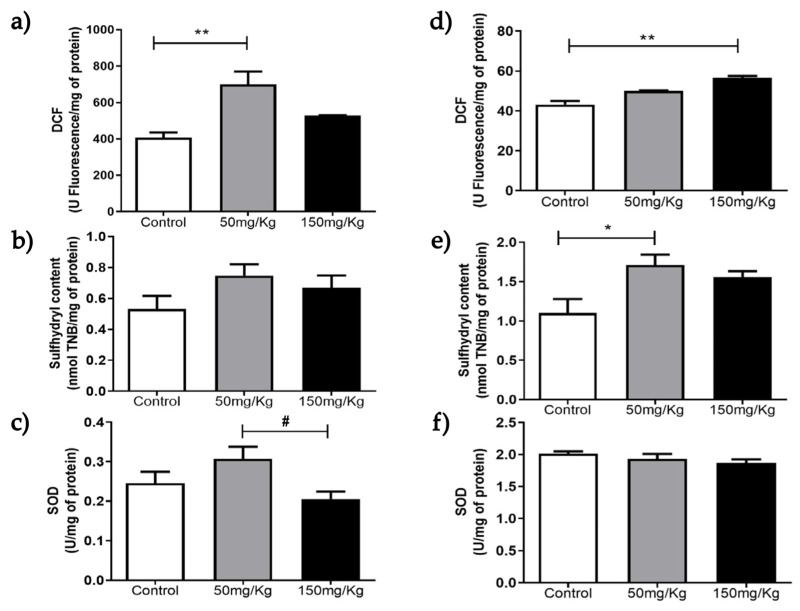
Analysis of DCF, sulfhydryl content and SOD levels 24 h (**a**–**c**) and 72 h (**d**–**f**) after IP administration of MEL-B at concentrations of 50 mg/kg and 150 mg/kg in mouse kidneys. * *p* < 0.05 and ** *p* < 0.005 when compared to the control group, and ^#^ *p* < 0.05 compared to the 150 mg/kg group, according to One-way ANOVA followed by Tukey’s Multiple Comparison Test. Each group at different concentrations and time points was analyzed from n = 6 samples.

**Figure 5:**
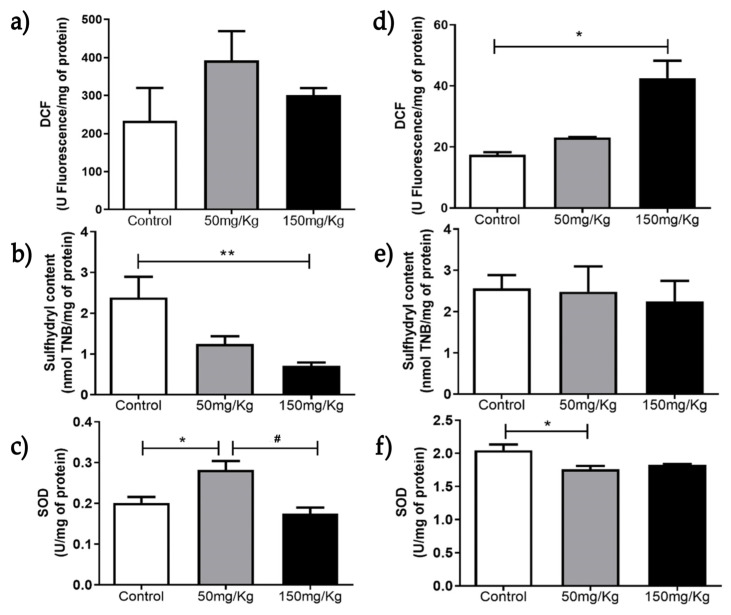
Analysis of DCF, sulfhydryl content, and SOD levels 24 h (**a**–**c**) and 72 h (**d**–**f**) after IP administration of MEL-B at concentrations of 50 mg/kg and 150 mg/kg in cardiac tissue. * *p* < 0.05 and ** *p* < 0.005 when compared to the control group, and ^#^ *p* < 0.05 compared to the 150 mg/kg group, according to One-way ANOVA followed by Tukey’s Multiple Comparison Test. Each group at different concentrations and time points was analyzed from n = 6 samples.

**Figure 6:**
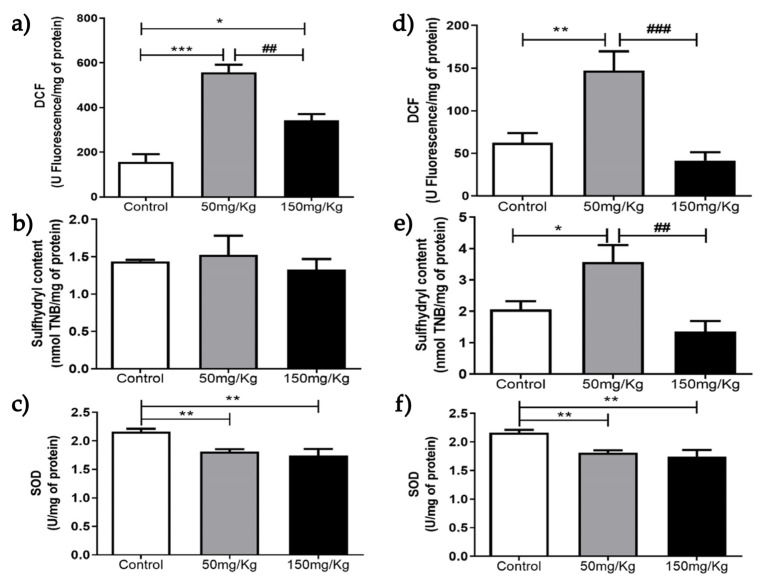
Analysis of DCF, sulfhydryl content, and SOD levels 24 h (**a**–**c**) and 72 h (**d**–**f**) after IP administration of MEL-B at concentration of 50 mg/kg and 150 mg/kg in mouse gastrocnemius muscles. * *p* < 0.05 ** *p* < 0.005, and *** *p* < 0.0005 when compared to the control group, and ^##^ *p* < 0.005 and ^###^ *p* < 0.0005 compared to the 150 mg/kg group, according to One-way ANOVA followed by Tukey’s Multiple Comparison Test. Each group at different concentrations and time points was analyzed from n = 6 samples.

Figure 1, Figure 2, Figure 3, Figure 4, Figure 5 and Figure 6 present the oxidants, oxidative damage, and antioxidant defense data for tissue samples from animals treated with two concentrations of MEL-B (50 and 150 mg/kg) administered for 24 and 72 h. Oxidant levels were measured as DCF, oxidative damage as sulfhydryl content, and antioxidant defenses as SOD activity. The animals in the control group were treated with PBS solution.

In the spleen tissue, over 24 h (Figure 1a), DCF levels were reduced (*p* < 0.05) compared to the untreated group, whereas after 72 h (Figure 1d), an increase in DCF content was observed at a dose of 150 mg/kg. There was no significant difference between the sulfhydryl content of samples (Figure 1b) after treatment with MEL-B for 24 h. An increase in sulfhydryl activity was observed for both doses tested (Figure 1e) after 72 h of IP administration. The groups exposed to MEL-B differed statistically from the PBS-treated (control) group, except for sulfhydryl content (Figure 1). The SOD levels showed a significant reduction in the spleen (Figure 1c–f) for both doses and exposure times (24 and 72 h), with a more pronounced effect at 24 h.

Treatments had the same influence on DCF levels for lung samples (Figure 2a,d). The sulfhydryl content decreased (*p* < 0.05) and increased (*p* < 0.05) after treatments with 150 mg/kg/24 h (Figure 2b) and 50 mg/kg/72 h, respectively. SOD activity was significantly lower at both MEL-B concentrations (*p* < 0.05) after 24 h of administration. No significant differences were observed in SOD activity at 72 h (Figure 2c,f).

Figure 3a shows that MEL-B influenced the level of DCF in liver samples compared to untreated ones when incubated for 24 h. Interestingly, when the glycolipid was administered for 72 h (Figure 2d), the treated samples differed from the control (*p* < 0.05) and each other (*p* < 0.05). Regarding sulfhydryl content, both treatments decreased sulfhydryl levels at 24 h (*p* < 0.05), with the effect more pronounced with 150 mg/kg of MEL-B (Figure 3b). No significant difference in sulfhydryl content was observed 72 h after administration of MEL-B (Figure 3e). SOD activity decreased (*p* < 0.05) for both treated groups at 24 h (Figure 3c). Figure 3f suggests that lung tissue treated for 72 h with 50 mg/kg of MEL-B was similar to the control; a significant decrease was observed only at 150 mg/kg.

In kidney samples, a significant increase (*p* < 0.05) in DCF levels was observed under the treatment conditions of 50 mg/kg/24 h (Figure 4a) and 150 mg/kg/72 h (Figure 4d). Sulfhydryl levels were only altered after administration of MEL-B at 50 mg/kg for 72 h (Figure 4b,e). The glycolipid concentrations modified SOD levels differently after 24 h of treatment (Figure 4c), but were similar to those in the sample without MEL-B. No alterations were observed in SOD activity at 72 h (Figure 4f).

The DCF levels of cardiac tissue were not altered after 24 h of IP administration (Figure 5a). For samples of the animals in which MEL-B was administered for 72 h, 150 mg/kg influenced the DCF levels (Figure 5d), while 50 mg/kg had no effect (*p* < 0.05). Regarding sulfhydryl content, there was a significant decrease (*p* < 0.05) at a dose of 150 mg/kg after 24 h of IP administration (Figure 5b). At 72 h, no significant differences were observed (Figure 5e). After 24 h of MEL-B administration, the SOD activity was significantly higher (*p* < 0.05) at a dose of 50 mg/kg compared to 150 mg/kg and the control group (Figure 5c). The animal samples treated with 50 mg/kg/72 h (Figure 5f) differed from the control, showing lower SOD activity.

Figure 6a reveals that treatment of gastrocnemius samples of animals with MEL-B/24 h promoted differences in DFC levels. At 50 mg/kg, the glycolipid led to the elevation of DFC levels (*p* < 0.05). Similar behavior was observed in the animal samples after 72 h of administration, except that the control and the 150 mg/kg dose showed the same effect (*p* < 0.05). MEL-B’s effects on the sulfhydryl content were observed only after 72 h (Figure 6b,e) of administration, where sulfhydryl levels were significantly higher (*p* < 0.05) at a dose of 50 mg/kg. As observed in Figure 6c,f, SOD activity decreased (*p* < 0.05) for animals treated with 50 and 150 mg/kg of MEL-B.

Table 1 summarizes the data from Figure 1 to Figure 6, showing the DCF, SOD, and sulfhydryl content of the gastrocnemius, liver, lung, kidney, heart, and spleen tissues, with and without MEL-B administration.

**Figure 7:**
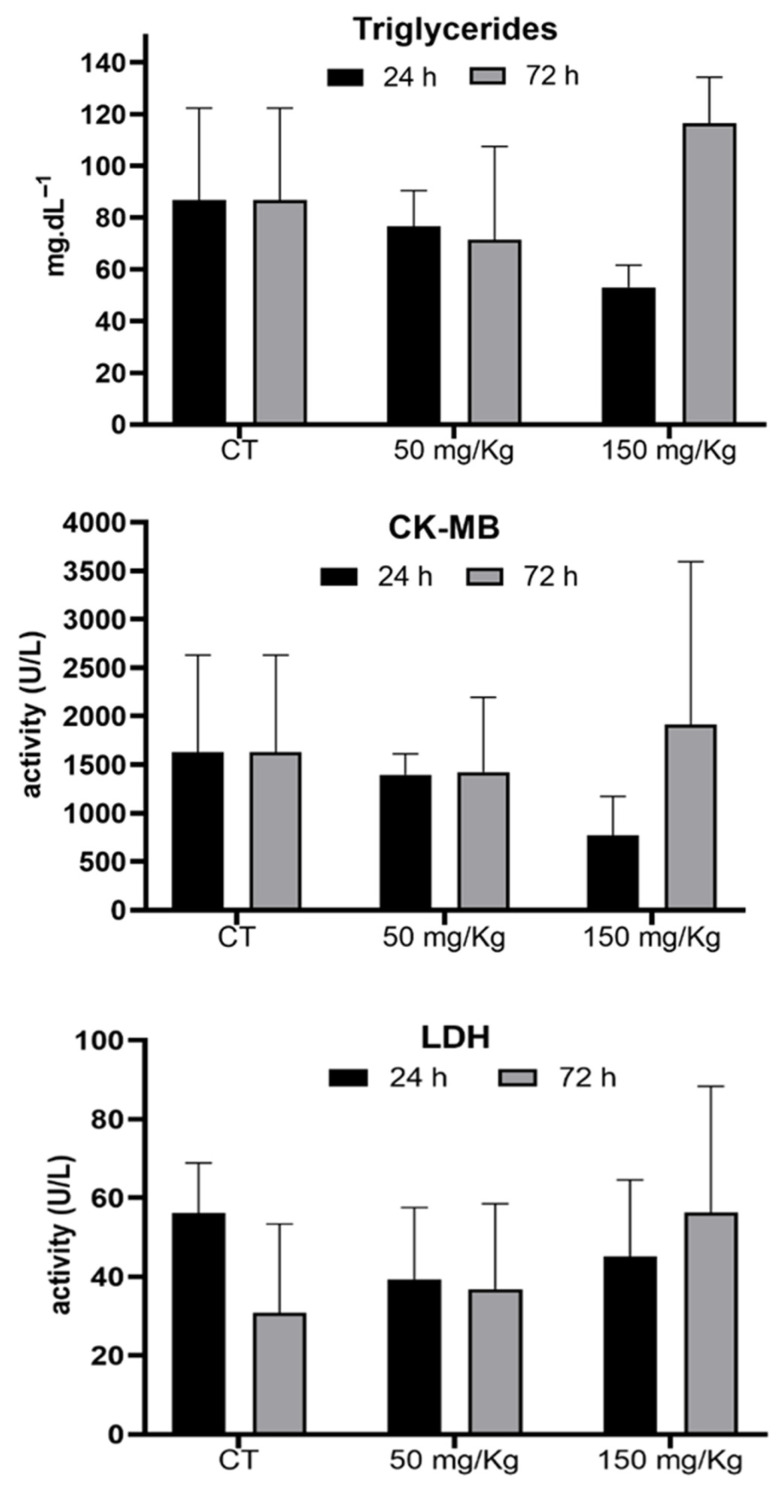
Analysis of triglyceride, CK-MB and LDH enzyme, and sulfhydryl levels 24 and 72 h after IP administration of MEL-B at concentrations of 50 (n = 6) and 150 mg/kg in the serum of mice (n = 6). No significant differences were observed according to One-way ANOVA followed by Tukey’s Multiple Comparison Test, when compared to the control group (n = 6).

Figure 7 shows the effect of MEL-B doses (50 and 150 mg/kg) on the triglyceride, CK-MB, and LDH levels. No significant differences were observed between samples of animals treated with and without the glycolipid.

## 4. Discussion

MELs have attracted attention from the cosmetic and pharmaceutical industries due to their non-toxicity and strong interactions with human skin, particularly with the deepest epidermal layer. MEL-B can be arranged in a lamellar phase, a structure similar to the stratum corneum’s molecular arrangement, and this structure also plays a role in tumor cells’ deregulation. Previous in vitro results showed the cytotoxic effect of MEL-B against B16F10 tumor cells [5,6]. Despite its reported properties, the oxidative levels and antioxidant defense effects of MEL-B in mammalian cells are unknown and poorly investigated.

Oxidative stress comes from an imbalance between oxidizing and antioxidant defenses. Therefore, oxidative damage occurs and promotes the oxidation of biomolecules [27,28]. When ROS exceed detoxification capacities, oxidative damage to lipids, nucleic acids, and proteins can occur. If this damage is irreparable, the cells will die by apoptosis (programmed cell death) or necrosis, depending on the extent of the damage. These mechanisms can result in the loss of enzyme or protein properties [29].

From spleen tissue analysis, it was concluded that 50 mg/kg of MEL-B/24 h does not promote ROS generation, suggesting its non-toxicity properties in mammals. The reduction in SOD activity indicates that glycolipids can exhibit an antioxidant effect, as DCF and sulfhydryl levels were not affected. This behavior was more evident for the 150 mg/kg dose, where the DCF levels were even lower. After 72 h of MEL-B administration, oxidant parameters indicate increased ROS production and decreased antioxidant defense at 150 mg/kg. Regarding sulfhydryl levels, the results suggest no effect on protein damage. Moreover, the data set indicates that 150 mg/kg of glycolipid induced ROS production, which is not a condition that can trigger oxidative stress.

In the lungs, DCF levels indicate that no ROS were produced after MEL-B administration. In addition, a dose of 50 mg/kg suggests an antioxidant effect of glycolipids. On the contrary, oxidative stress was evidenced in liver samples. The liver is an organ involved in several cycles of metabolizing harmful biological compounds. Therefore, even low concentrations of specific molecules (e.g., food or drugs) can trigger ROS production. The data from liver assays suggest that long periods (72 h) are required for full metabolization of biosurfactants. Similar results were reported by Feuser et al. [6], who evaluated the acute toxicity of polymeric nanoparticles. From this research, the authors demonstrated that the synthesized material promoted alterations in the sulfhydryl and SOD levels in the liver, suggesting that the glutathione route is an alternative for counteracting the nanoparticles’ effects.

The kidney assays showed that the lower MEL-B dose (50 mg/kg) induced ROS production after 24 h of administration. No alterations were observed in antioxidant defenses and protein damage. After 72 h, the concentration of 150 mg/kg of MEL-B induced ROS production, while the dose of 50 mg/kg did not produce any alterations. The results showed that both DCF and SOD content increased, culminating in an oxidative balance [28].

The analysis of cardiac tissue showed that MEL-B has an antioxidant effect at a dose of 50 mg/kg. On the other hand, a concentration of 150 mg/kg induced oxidative stress, as evidenced by increased ROS levels. Furthermore, the concentration of 150 mg/kg also caused oxidative damage, as shown by the sulfhydryl levels.

MEL-B induced ROS production at 24 and 72 h in the gastrocnemius muscle. A specific condition of 150 mg/kg MEL-B/72 h reduced the antioxidant levels with no alteration in ROS production, suggesting an antioxidant effect of the glycolipid. In addition, the sulfhydryl levels suggest no protein damage in this tissue.

In general, in the analysis of tissues/organs, it can be assumed that MEL-B presents antioxidant activity, mainly at a concentration of 50 mg/kg. Liver samples were an exception to this behavior, as the data indicated oxidative cell damage.

Radical-scavenging activity of MEL-B was reported by Takahashi et al. [15]. The authors suggest that the radical-scavenging activity of MEL-B is related to its arrangement. Radical scavenging was more evident when MEL-B was in free form, while superoxide anion-scavenging was observed for the self-assembled structure. These data are aligned with our findings since MEL-B showed scavenging activity for free radicals, but no superoxide anion activity was observed. The liver and spleen tend to have high superoxide anion levels, which may explain the lack of reduction in DCF levels in these organs.

Morita et al. [30] showed that MELs decrease oxidative damage of cells by reducing ROS production. A similar result was obtained for almost all structures analyzed in the current study. The spleen samples were an exception, as significant increases in DCF levels and decreases in thiol groups were observed after cells were exposed to 150 mg/kg of MEL-B for 72 h. The results for the other organs agree with the literature data, which have demonstrated that MELs have no adverse effect on the cells, and their antioxidant effect can be due to free-radical scavenging [15,30].

Triglyceride levels were a systemic marker of metabolic and hepatic perturbation in toxicological assessments. Alterations in circulating triglycerides are commonly associated with early disturbances in lipid metabolism, mitochondrial function, and hepatic homeostasis, which may occur even in the absence of histopathological damage during short-term exposure [31,32]. However, no alterations were observed in the activity of LDH and CK-MB enzymes, indicating the lack of tissue damage evaluated by these parameters, even in the presence of oxidative stress in the liver.

The data presented in the current study show that MEL-B did not trigger consistent alterations in gastrocnemius, lung, kidney, cardiac, or pulmonary tissue when administered intraperitoneally, suggesting the absence of marked tissue dysfunction under the experimental conditions tested. Previous findings showed that MEL-B presented promising cytotoxic results in cancer cells, whereas an innocuous effect was observed in fibroblasts [5,6,15,30]. Moreover, MEL-B can cross cell membranes, increasing ROS levels; however, enzymatic activity is not compromised, indicating reversible oxidative damage [15,30]. Thus, the current results open new perspectives for the investigation of the cosmetic, therapeutic, and pharmacological properties of MEL-B.

Although MEL-B is commonly described as a biodegradable and biocompatible biosurfactant with many pharmacological, food, and biomedical applications, a significant gap remains in the literature regarding its safety for in vivo applications [33]. Only a limited number of studies have investigated the acute toxicity of MEL-B in living organisms, and even fewer have addressed its potential chronic toxicity [18,19,34]. This lack of comprehensive in vivo toxicological data represents a significant knowledge gap that needs to be addressed before broader biological or medical applications can be fully supported. In this context, the present study constitutes an initial step toward elucidating the effects of MEL-B exposure in vivo, providing data to support and guide future investigations into long-term toxicity and safety.

Future studies should expand the toxicological evaluation of MEL-B by investigating alternative routes of administration and more prolonged exposure periods, allowing a more comprehensive assessment of its biological effects. Additionally, studies including animals with different pathological conditions may provide relevant insights into how metabolic status influences responses to MEL-B. Considering the current findings on oxidative stress markers, particular attention should be directed toward the liver, which appears more sensitive to MEL-B exposure and warrants deeper mechanistic and functional investigation.

## 5. Conclusions

This study addresses preliminary toxicological effects of MEL-B administration in Swiss mice, analyzing reactive oxygen species, oxidative damage markers, antioxidant defenses, protein content, triglycerides, and CK-MB, and LDH levels in the gastrocnemius muscle, liver, lungs, kidneys, cardiac tissue, and spleen. There was no evidence of significant alteration following exposure to MEL-B in the gastrocnemius muscle, lungs, kidneys, cardiac tissue, and spleen, at either 50 mg/kg or 150 mg/kg doses and at 24 h or 72 h. However, ROS production and oxidative stress were detected specifically in liver samples, indicating that hepatic tissue is more sensitive than the other organs analyzed. These findings suggested that, under the experimental conditions evaluated, MEL-B did not trigger significant alterations in the organs analyzed, reinforcing its biocompatibility while highlighting the liver as a relevant target organ for further investigation. To gain a more comprehensive understanding, future studies should address the effects of MEL-B over extended exposure periods, exploring alternative administration routes, and using animal models with different pathologies. The outcome of this study provides important support for further consideration of safe dosing regimens and target organs, and highlights the potential for MEL-B’s application in therapeutic, pharmacological, and medical research settings.

## Figures and Tables

**Table 1:** Different biochemical parameters (DCF, sulfhydryl, and SOD) were evaluated after the IP administration of MEL-B at doses of 50 mg/kg and 150 mg/kg.

50 mg/kg—24 h				50 mg/kg—72 h			
Organs	DCF	Sulfhydryl	SOD		DCF	Sulfhydryl	SOD
Spleen	=	=	-	Spleen	=	+	--
Lung	=	=	-	Lung	=	+	=
Liver	+	+	--	Liver	+++	=	=
Kidney	++	=	=	Kidney	=	+	=
Heart	=	=	+	Heart	=	=	-
Gastrocnemius	+++	=	--	Gastrocnemius	++	+	--
**150 mg/kg—24 h**				**150 mg/kg—72 h**			
Organs	DCF	Sulfhydryl	SOD		DCF	Sulfhydryl	SOD
Spleen	-	=	-	Spleen	++	++	--
Lung	=	-	-	Lung	=	=	=
Liver	++	+++	--	Liver	+	=	-
Kidney	=	=	=	Kidney	++	=	=
Heart	=	--	=	Heart	+	=	=
Gastrocnemius	+	=	--	Gastrocnemius	=	=	--

+: increase of *p* < 0.05; ++: increase of *p* < 0.005; +++: increase of *p* < 0.0005; -: decrease of *p* < 0.05; --: decrease of *p* < 0.005; =: no statistical difference (all compared to the control group, according to One-way ANOVA followed by Tukey’s Multiple Comparison Test).

## Data Availability

The original contributions presented in this study are included in the article. Further inquiries can be directed to the corresponding author.

## Abbreviations

The following abbreviations are used in this manuscript:

MEL-B	Mannosylerythritol lipid-B
CK-MB	Creatine kinase
LDH	Lactate dehydrogenase
DCF	2′,7′-dichlorofluorescein
SOD	Superoxide dismutase
MEL	Mannosylerythritol lipid
MEL-A	Mannosylerythritol Lipid-A
MEL-C	Mannosylerythritol Lipid-C
RBL-2H3	Rat basophilic leukemia cell line
TNF-α	Tumor necrosis factor
SNARE	Soluble N-ethylmaleimide-sensitive factor attachment protein receptor
MAP	Mitogen-activated protein kinase
DCFH-DA	2′,7′-dichlorodihydrofluorescein diacetate
PBS	Sodium phosphate buffer
ROS	Reactive oxygen species

## References

[1] Arutchelvi J.I., Bhaduri S., Uppara P.V., Doble M. (2008). Mannosylerythritol lipids: A review. J. Ind. Microbiol. Biotechnol..

[2] Beck A., Werner N., Zibek S. (2019). Mannosylerythritol Lipids: Biosynthesis, Genetics, and Production Strategies. Biobased Surfactants.

[3] Niu Y., Wu J., Wang W., Chen Q. (2019). Production and characterization of a new glycolipid, mannosylerythritol lipid, from waste cooking oil biotransformation by *Pseudozyma aphidis* ZJUDM34. Food Sci. Nutr..

[4] de Andrade C.J., Coelho A.L., E Feuser P., de Andrade L.M., Carciofi B.A., de Oliveira D. (2022). Mannosylerythritol lipids: Production, downstream processing, and potential applications. Curr. Opin. Biotechnol..

[5] Coelho A.L.S., Feuser P.E., Carciofi B.A.M., de Andrade C.J., de Oliveira D. (2020). Mannosylerythritol lipids: Antimicrobial and biomedical properties. Appl. Microbiol. Biotechnol..

[6] Feuser P.E., Coelho A.L.S., de Melo M.E., Scussel R., Carciofi B.A.M., Machado-De-Ávila R.A., de Oliveira D., de Andrade C.J. (2021). Apoptosis Induction in Murine Melanoma (B16F10) Cells by Mannosylerythritol Lipids-B; a Glycolipid Biosurfactant with Antitumoral Activities. Appl. Biochem. Biotechnol..

[7] de Andrade C.J., de Andrade L.M., Rocco S.A., Sforça M.L., Pastore G.M., Jauregi P. (2017). A novel approach for the production and purification of mannosylerythritol lipids (MEL) by Pseudozyma tsukubaensis using cassava wastewater as substrate. Sep. Purif. Technol..

[8] Liu X., Shu Q., Chen Q., Pang X., Wu Y., Zhou W., Wu Y., Niu J., Zhang X. (2020). Antibacterial efficacy and mechanism of mannosylerythritol lipids-A on *Listeria monocytogenes*. Molecules.

[9] Shu Q., Wei T., Liu X., Liu S., Chen Q. (2022). The dough-strengthening and spore-sterilizing effects of mannosylerythritol lipid-A in frozen dough and its application in bread making. Food Chem..

[10] Silva I.A., Veras B.O., Ribeiro B.G., Aguiar J.S., Guerra J.M.C., Luna J.M., Sarubbo L.A. (2020). Production of cupcake-like dessert containing microbial biosurfactant as an emulsifier. PeerJ.

[11] Shu Q., Niu Y., Zhao W., Chen Q. (2019). Antibacterial activity and mannosylerythritol lipids against vegetative cells and spores of *Bacillus cereus*. Food Control.

[12] Fan L., Li H., Niu Y., Chen Q. (2016). Characterization and inducing melanoma cell apoptosis activity of mannosylerythritol lipids-A produced from *Pseudozyma aphidis*. PLoS ONE.

[13] Zhao X., Murata T., Ohno S., Day N., Song J., Nomura N., Nakahara T., Yokoyama K.K. (2001). Protein Kinase Cα Plays a Critical Role in Mannosylerythritol Lipid-induced Differentiation of Melanoma B16 Cells. J. Biol. Chem..

[14] Wakamatsu Y., Zhao X., Jin C., Day N., Shibahara M., Nomura N., Nakahara T., Murata T., Yokoyama K.K. (2001). Mannosylerythritol lipid induces characteristics of neuronal differentiation in PC12 cells through an ERK-related signal cascade. Eur. J. Biochem..

[15] Takahashi M., Morita T., Fukuoka T., Kitamoto D. (2012). Glycolipid Biosurfactants, Mannosylerythritol Lipids, Show Antioxidant and Protective Effects against H_2_O_2_-Induced Oxidative Stress in Cultured Human Skin Fibroblasts. J. Oleo Sci..

[16] Bae I., Lee E.S., Yoo J.W., Lee S.H., Ko J.Y., Kim Y.J., Lee T.R., Kim D., Lee C.S. (2019). Mannosylerythritol lipids inhibit melanogenesis via suppressing ERK-CREB-MiTF-tyrosinase signalling in normal human melanocytes and a three-dimensional human skin equivalent. Exp. Dermatol..

[17] Bae I.-H., Lee S.H., Oh S., Choi H., Marinho P.A., Yoo J.W., Ko J.Y., Lee E.-S., Lee T.R., Lee C.S. (2019). Mannosylerythritol lipids ameliorate ultraviolet A-induced aquaporin-3 downregulation by suppressing c-Jun N-terminal kinase phosphorylation in cultured human keratinocytes. Korean J. Physiol. Pharmacol..

[18] Cheng X., Geng J., Wang L., Ma X., Su Y., Arif M., Liu C. (2023). Berberine-loaded mannosylerythritol lipid-B nanomicelles as drug delivery carriers for the treatment of Helicobacter pylori biofilms in vivo. Eur. J. Pharm. Biopharm..

[19] Liu X., Zhang L., Pang X., Wu Y., Wu Y., Shu Q., Chen Q., Zhang X. (2022). Synergistic antibacterial effect and mechanism of high hydrostatic pressure and mannosylerythritol Lipid-A on Listeria monocytogenes. Food Control.

[20] Rana S., Singh J., Wadhawan A., Khanna A., Singh G., Chatterjee M. (2021). Evaluation of In Vivo toxicity of Novel Biosurfactant from *Candida parapsilosis* loaded in PLA-PEG Polymeric Nanoparticles. J. Pharm. Sci..

[21] Liou G.-Y., Storz P. (2010). Reactive oxygen species in cancer. Free Radic. Res..

[22] Silva I., Alípio C., Pinto R., Mateus V. (2021). Potential anti-inflammatory effect of erythropoietin in non-clinical studies in vivo: A systematic review. Biomed. Pharmacother..

[23] Eruslanov E., Kusmartsev S. (2010). Identification of ROS using oxidized DCFDA and flow-cytometry. Methods Mol. Biol..

[24] Aksenov M.Y., Markesbery W.R. (2001). Changes in thiol content and expression of glutathione redox system genes in the hippocampus and cerebellum in Alzheimer’s disease. Neurosci. Lett..

[25] Bannister J.V., Calabrese L. (1987). Assays for Superoxide Dismutase. Methods Biochem. Anal..

[26] Lowry O.H., Rosebrough N.J., Farr A.L., Randall R.J. (1951). Protein measurement with the Folin phenol reagent. J. Biol. Chem..

[27] Cooke M.S., Evans M.D., Dizdaroglu M., Lunec J. (2003). Oxidative DNA damage: Mechanisms, mutation, and disease. FASEB J..

[28] Hernández-Ruiz Á., García-Villanova B., Guerra-Hernández E., Amiano P., Ruiz-Canela M., Molina-Montes E. (2019). A review of a priori defined oxidative balance scores relative to their components and impact on health outcomes. Nutrients.

[29] Kupsco A., Schlenk D. (2015). Oxidative Stress, Unfolded Protein Response, and Apoptosis in Developmental Toxicity. Int. Rev. Cell Mol. Biol..

[30] Morita T., Konishi M., Fukuoka T., Imura T., Kitamoto D. (2006). Discovery of Pseudozyma rugulosa NBRC 10877 as a novel producer of the glycolipid biosurfactants, mannosylerythritol lipids, based on rDNA sequence. Appl. Microbiol. Biotechnol..

[31] Amacher D., Schomaker S., Burkhardt J. (1998). The Relationship Among Microsomal Enzyme Induction, Liver Weight and Histological Change in Rat Toxicology Studies. Food Chem. Toxicol..

[32] Amacher D.E. (2012). The primary role of hepatic metabolism in idiosyncratic drug-induced liver injury. Expert Opin. Drug Metab. Toxicol..

[33] de Almeida J.D., Nascimento M.F., Keković P., Ferreira F.C., Faria N.T. (2024). Unlocking the Potential of Mannosylerythritol Lipids: Properties and Industrial Applications. Fermentation.

[34] Wu Y., Geng J., Cheng X., Yang Y., Yu Y., Wang L., Dong Q., Chi Z., Liu C. (2022). Cosmetic-Derived Mannosylerythritol Lipid-B-Phospholipid Nanoliposome: An Acid-Stabilized Carrier for Efficient Gastromucosal Delivery of Amoxicillin for In Vivo Treatment of *Helicobacter pylori*. ACS Omega.

